# Application of Textile Composite Materials as a Sorbent for Cleaning Up Oil Spills

**DOI:** 10.3390/ma18051146

**Published:** 2025-03-04

**Authors:** Daniela Angelova, Desislava Staneva, Daniela Atanasova, Vesislava Toteva

**Affiliations:** Department of Textile, Leather and Fuels, University of Chemical Technology and Metallurgy, 8 Kliment Ohridski Blvd., 1797 Sofia, Bulgaria; d.atanasova1@abv.bg (D.A.); vesislava@uctm.edu (V.T.)

**Keywords:** oil spill, textile composite materials, sorption, regeneration

## Abstract

This article compares two new textile materials used to clean up spills of oil or two oil products (crude oil, diesel fuel, and base oil SN 150). The plain-woven cotton fabric is hydrophilic, with a typical porous structure. After coating with a layer of chitosan modified with benzaldehyde and cross-linked with glutaraldehyde (CB), its hydrophobicity increases, hence the sorption affinity to hydrophobic hydrocarbons. Including in situ synthesized zinc oxide particles in the hydrophobic chitosan layer (CBZ) changes its structure and increases the sorption capacity. The morphology of the layers was assessed using scanning electron microscopy (SEM) and by comparing the contact angles of the pollutants against the cotton fabric and the composite materials. EDX analysis and mapping for the Zn element show that zinc is homogeneously distributed on the fabric surface. The roughness enhancement and mesoporous structure under the influence of zinc oxide particles were established by the Brunauer Emmett Teller (BET) method and atomic force microscopy (AFM). The advantages of textile composites are their flexibility, stability, and ability to float on the water and wipe up oil spills. It was found that the materials can be successfully regenerated and used repeatedly, making them highly effective because the sorbed crude oil or petroleum products can be separated and utilized.

## 1. Introduction

The pollution of the hydrosphere with oil and oil products leads to profound, in most cases irreversible, changes in the chemical, physical, and microbiological properties of ecosystems. In addition to harmful effects on human and animal health, oil pollution causes long-term losses to several economic sectors, such as agriculture, fishing, and tourism [1]. These facts determine the relevance of research in developing strategies and means for predicting and liquidating oil spill pollution [2]. Spills of crude oil and oil products in the seas and oceans can be removed by various methods, grouped as mechanical, physicochemical, biological, and photocatalytic. Using sorbents as a physicochemical method for removing oil spills is among the most effective [3].

The choice of sorbent depends on several factors. Its main objective is to limit the adverse impact of the event by quickly and completely containing the spill. Therefore, whichever sorbent is currently available is immediately used. Thus, the sorbent must be pre-selected and convenient for storage. Once the situation is assessed, factors such as the size and type of spill, water availability, how the sorbent will be transported, efficiency and ease of use become critical. Next, other features become essential, such as the cost of the sorbent, its ability to regenerate, and how it will be disposed of because it can become a secondary source of environmental pollution.

One of the directions related to solving this problem is the development of new effective sorbents from cheap and accessible raw materials [4,5,6,7]. Increasingly, scientific interest is directed towards using waste materials, such as agricultural or industrial waste, including textiles, as cheap sorbents [8].

Natural fibrous organic materials such as cotton fiber, corn stalk, and non-woven fabric (wool) are affordable and environmentally friendly [9,10,11]. These sorbents provide rapid oil removal through the pore-filling mechanism, but their volume limits their sorption capacity. Another of their disadvantages is that they can get wet with water and oil at the same time, so their effectiveness is reduced. To overcome these drawbacks, it is necessary to develop new materials and technologies for the fast and efficient cleanup of oil spills. The sorption properties of these materials can be improved by modifying them with additionally functionalized natural polymers and metals or metal oxides [12]. As a result, organic–inorganic nanocomposite structures are attained.

The sorbent must be hydrophobic and oleophilic for fast and efficient cleanup of oil spills. The wetting behavior generally depends on surface chemistry (i.e., surface energy) and surface topography (i.e., physical roughness). It is commonly characterized by the contact angle of the liquid on the solid surface in the presence of another fluid (e.g., gas or another liquid). Three main theoretical models (Young, Wenzel, and Cassie-Baxter) describe the relationship between surface morphology and liquid. They try to explain the natural examples of super-wetting or non-wetting surfaces, inspiring the creation of green, environmentally friendly, and sustainable materials with biomimetic hierarchical surfaces [13].

A hydrophobic material can be obtained by coating it with a lower surface energy substance or by forming the specific rough surface microstructures designed to minimize solution wetting. The wettability of the surface is controlled at molecular level by the type, length, and shape of the functional groups. The presence of hydrocarbons with long aliphatic (linear) or branched chains or aromatic molecules, fluorocarbons, or silicone-based polymers decreases the surface energy and promotes hydrophobicity [14]. Surface morphology also exhibits a significant role in the wetting characteristics. It can be established by different chemical or physical methods [15].

One of the most widely used biopolymers as a sorbent is chitosan. It is biodegradable, environmentally friendly, inexpensive, and produced from renewable waste products [16,17]. It has an appropriate number of functional groups that participate in the formation of complexes with various metal ions, from which, after reduction, nanoparticles can be obtained. Different nanoparticles such as SiO_2_, ZnO, TiO_2_, and Fe_3_O_4_ have been used to improve the surface and increase the hydrophobicity of the materials [3,18,19]. Our previous study found that the interaction of ZnO particles with the functional groups of chitosan affects its swelling ability in the water, thus determining its sorption properties [20]. The ZnO particles form stable complexes with chitosan macromolecules’ hydrophilic hydroxyl and amino groups, further enhancing their hydrophobic properties. Studies of the surface wettability of ZnO nanostructures also confirm their hydrophobicity based on their surface morphology, size, and form [21]. Moreover, ZnO nanoparticles have biocompatibility and biodegradability, antimicrobial, and photocatalytic properties [22,23].

Developing recyclable sorbents is crucial to avoid secondary pollution during oil removal. The ability to recycle or reuse the materials after the sorption of oil and oil products leads to lower costs and is essential for the environment. There are several methods for recycling the sorbents: squeezing, heating, incineration, and vacuum filtration [24,25]. Solvent extraction is often used. The process consists of immersing the saturated sorbents in a suitable solvent to extract the oil and reuse the sorbents [26].

Our recent research has demonstrated that a material based on natural products, such as cotton fabric modified by chitosan cross-linked with glutaraldehyde and zinc oxide particles, exhibits many advantages, such as ease of use, regeneration, and reusability [20]. Since oil spills occur in water or wet areas, the effectiveness of the sorbent depends on its oleophilic and hydrophobic properties. Chitosan has three reactive functional groups: amino, hydroxyl, and acetamido. Replacing its hydrophilic groups with an aromatic substituent can readily enhance its hydrophobicity. Therefore, using aldehydes for chitosan modification will introduce new hydrophobic substituents, such as aromatic moieties, and provide greater strength to the formed film.

Benzaldehyde and glutaraldehyde can also be considered environmentally friendly products. The first naturally occurs in many nuts, vegetables, and cheeses and is used as a food flavor, and the second is widely used as a high-level disinfectant and chemical sterilant in the medical field [27,28].

The aim of this research is to obtain composite textile materials by amending hydrophilic cotton fabric with chitosan modified with benzaldehyde and glutaraldehyde. The incorporation of ZnO nanoparticles in the attained hydrophobic layer will increase the sorption capacity for oil and oil products. The materials’ surfaces were established by optical and scanning electron microscopy and contact angle comparison. The specific surface area, roughness, and porosity of the materials were examined by the Brunauer Emmett Teller method and atomic force microscopy. The potential for regeneration and the multiple uses of the obtained composite textile materials were evaluated, providing an outlook on the practical applications of the research.

## 2. Materials and Methods

### 2.1. Materials

The experiments were carried out with 100% cotton fabric, plain woven with a weight per unit area of 135 ± 5 g/m^2^. The reagents used to modify the starting material are chitosan, with a molecular weight of 600,000–800,000 (ACROS organics; Geel, Belgium); glacial acetic acid, benzaldehyde, and glutaraldehyde (25% aqueous solution) (MERCK; Darmstadt, Germany); zinc nitrate Zn(NO_3_)_2_ × 6H_2_O, and NaOH (Valerus Ltd.; Sofia, Bulgaria). Distilled water and ethanol were used as solvents.

Crude oil (Arabian Light, Ahmadi, Kuwait), diesel fuel (Neftohim Burgas AD, Burgas, Bulgaria), and base oil SN 150 (Prista Oil, Ruse, Bulgaria) were used to study the sorption properties of the composite textile materials. The characteristics of the sorbates are given in Table 1.

### 2.2. Preparation of the Sorbent Materials

**Figure 1:**
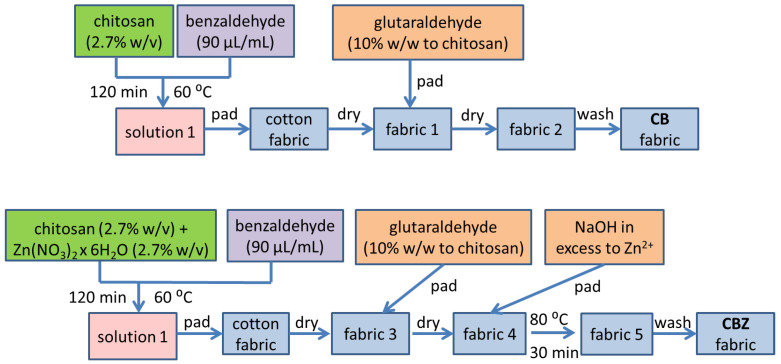
Schematic representation of the CB and CBZ sorbent materials’ preparation.

The cotton fabric was modified according to a methodology described in a previous study [31]. Figure 1 shows a schematic representation of the CB and CBZ sorbent materials’ preparation. A sample of CB was obtained by the pad–dry–pad–dry–wash method. The cotton fabric was soaked in a solution of chitosan modified with benzaldehyde. After drying, the fabric was treated with a glutaraldehyde water solution and dried at room temperature for 24 h. It was rinsed with distilled water and left to dry in ambient air.

A sample of CBZ was obtained using the following method: pad–dry–pad–dry–pad (alkali)–cure–wash. The cotton fabric was impregnated with the chitosan solution, modified with benzaldehyde, and contained zinc ions. After drying, the fabric was treated with a glutaraldehyde water solution and dried at room temperature for 24 h. Subsequently, the CBZ sample was immersed in a sodium hydroxide solution with an excess of ten times the stoichiometric amount of zinc ions and heated at 80 °C for 30 min. The resulting composite materials were washed with distilled water and left to dry at room temperature.

### 2.3. Analysis

The surface structure of the composite materials and the development of ZnO particles were examined using a SEM-EDX device (SEM/FIB LYRA I XMU SEM (TESCAN, Brno, Czech Republic)) with a tungsten heating filament. This equipment offered a resolution of 3.5 nm at 30 kV and operated within an accelerating voltage range of 200 V to 30 kV. Before imaging, the samples were coated with gold using a DC magnetron sputtering system Au K500X (Quorum Technologies, Lewes, UK).

The pristine cotton fabric and CB and CBZ samples were examined by low-temperature nitrogen physisorption with a Quantachrome Instruments AUTOSORB iQ-C-MP-AG-AG NOVA 1200e (Ashland, VA, USA). The specific surface area is determined by the Brunauer Emmett Teller equation (BET).

The surface roughness of cotton fabric before and after its modification was measured by atomic force microscopy (AFM, Bruker Nano GmbH, Berlin, Germany).

The contact angles of the crude oil, diesel fuel, and base oil SN 150 on the pristine cotton fabric and CB and CBZ composites were measured with a THETA Flow Auto 1 Optical Tensiometer and Basler acA-2500-60 µm digital camera (Biolin Scientific AB, Gothenburg, Sweden). The reported results represent the average value of five measurements.

### 2.4. Sorption Studies

#### 2.4.1. Measurment of Sorption Capacity

Sorption studies were carried out by immersing the samples in 200 mL water and 10 mL crude oil, diesel, or base oil, respectively, for 10 min. After draining the excess oil products, the sorbed amount is determined as the difference between the weight of the wet material (*m*_2_) and its initial weight (*m*_1_). The sorption capacity of the obtained composite materials was calculated using Equation (1). The water sorption capacity of the cotton fabric and the samples was conducted and calculated using the same methodology and equation. Each assay was performed at least three times at room temperature.

(1) $$Sorption\;capacity=\frac{{m_{2}-m}_{1}}{m_{1}},\;{g.g}^{-1}\;$$

#### 2.4.2. Material Regeneration

The regeneration of the CBZ material and its multiple uses were investigated. The experiment was conducted by placing the sample in 200 mL water and 10 mL crude oil or oil product for 3 min. After removal, the material was drained from excess oil products for one minute and weighed. The regeneration was performed by immersion of the sample in n-hexane for 3 min. The sample was used numerous times until the spill was removed entirely from the water. The percentage sorbed amount was calculated by Equation (2), where *w*_1_ (g) and *w*_2_ (g) represent the total and sorbed amount of the spill, respectively.

(2) $$Spill\;removal=\frac{w_{2}}{w_{1}}*100,\;\%$$

## 3. Results and Discussion

### 3.1. SEM Analysis

**Figure 2:**
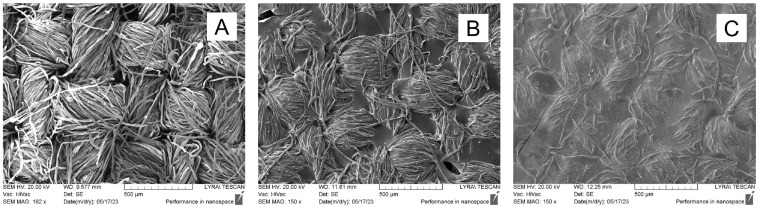
SEM micrographs of (**A**) cotton fabric; (**B**) CB fabric; (**C**) CBZ fabric.

SEM analysis was used to determine the change in the surface structure of the obtained materials after their modification. Figure 2A shows the characteristic construction of the cotton fabric obtained during its plain weaving, in which the individual cotton fibers were twisted into a yarn that was interwoven in a certain way. As a result, gaps of different sizes were obtained in the fabric structure. The formation of a film on the fabric surface was observed when the benzaldehyde-modified chitosan layer was applied and with its subsequent cross-linking with glutaraldehyde. This can be seen in Figure 2B. It binds the individual fibers of the yarn and fills the gaps between the individual threads perpendicular to each other. The inclusion of zinc oxide particles, in the applied layer, leads to a denser surface coating, in which the characteristic relief of the investigated cotton fabric is almost lost (Figure 2C).

**Figure 3:**
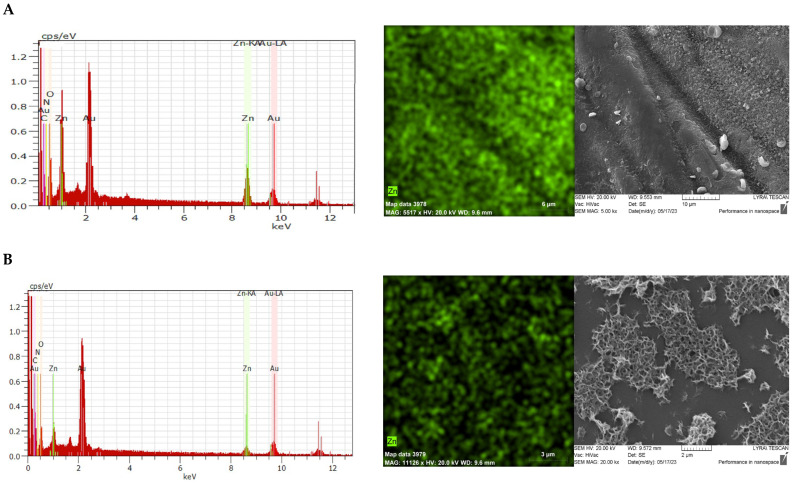
EDX analysis, EDX mapping for Zn element distribution and SEM micrographs of CBZ (**A**) on the yarn surface and (**B**) in the void space between the yarns.

EDX analysis and mapping for the Zn element show that zinc is homogeneously distributed on the fabric surface, but its content is higher on the top of the yarn than in the film formed in the void between the yarns (Figure 3).

The ability of zinc ions to complex with the functional groups of chitosan changes the structure of the resulting layer on the fabric. As a result of sodium hydroxide treatment and subsequent heat treatment, star-shaped zinc oxide particles were formed. A specific continuous network with a very well-developed surface is formed (Figure 3A). When observing the surface between the fibers, it can be seen that the zinc oxide particles form numerous islands surrounded by the chitosan layer but retain their characteristic well-developed surface (Figure 3B). It can be assumed that this specific rough surface is the reason for the improved sorption properties of the obtained material in relation to oil and oil products.

**Figure 4:**
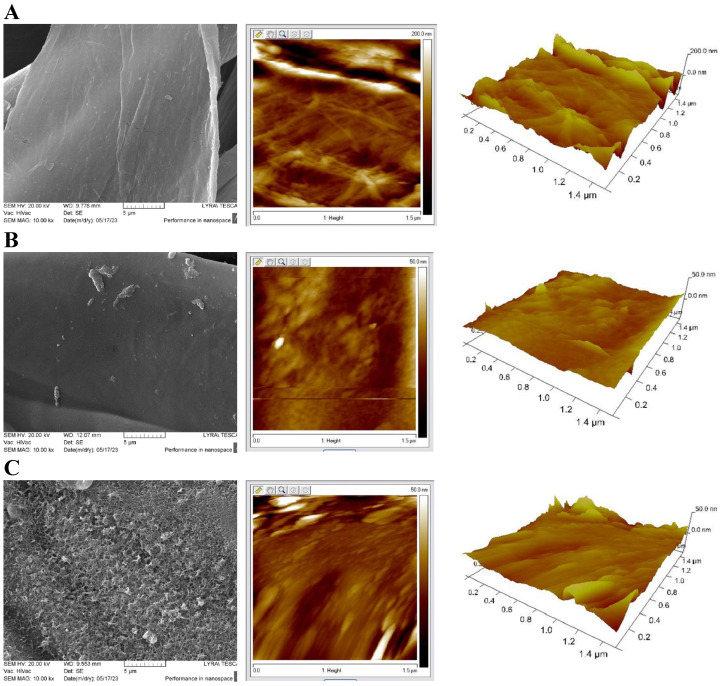
SEM images at 10.00k× magnification and AFM 2D and 3D images of (**A**) cotton fibers; (**B**) CB fibers; (**C**) CBZ fibers.

Figure 4 compares SEM images with 2D and 3D AFM images of cotton fiber surfaces and the modified fibers of samples of CB and CBZ. Raw cotton fibers have a typical surface morphology characterized by fibrils aligned at a certain angle to the fiber axis (Figure 4A). When a layer of chitosan modified with aldehydes is applied in material CB, a relatively smooth fiber surface is obtained, but it can be seen that the formed film is wrinkled, and agglomerates with different sizes are unevenly located on it (Figure 4B). The presence of zinc oxide particles in the film significantly changes the surface in material CBZ. The zinc oxide particles in the chitosan film form a highly porous visible layer, as seen in the SEM image. AFM analysis shows a slightly rough surface morphology with a granular structure and relatively small grain size (Figure 4C).

### 3.2. Interaction of Crude Oil and Diesel Fuel or Base Oil SN 150 Droplets on Cotton Fabric, CB, and CBZ

Contact angle measurements provide insights into the liquid wetting properties and its adhesion to the material. Modifying chitosan with aromatic moieties improves oil adhesion and water repulsion. The Wenzel model suggests that embedding ZnO particles in the chitosan film and increasing the surface roughness raise the contact angle of water on hydrophobic surfaces. Conversely, increased roughness enhances the specific surface area and provides more adsorbed oil pollutant molecules [15]. To compare the interaction of crude oil and its derivates on pristine cotton fabric (CO) and the obtained CB and CBZ composite materials, a droplet of each of them was placed onto the material surface using a syringe. The acquired results are summarized in Table 2 and Figure S1. The contact angle of crude oil and base oil decreases in the same order: cotton fabric > CB > CBZ. These results confirm that deposition of the chitosan layer, containing aromatic units, enhanced the hydrophobicity of the fabric surface and favored the interaction with oil pollutants. Including ZnO particles in this layer creates sorbent pores that increase oil attachment and increase the entrapment rate. Since the viscosity of the base oil is higher than that of crude oil, it is more difficult to enter the pores, and the rate of sorption decreases. On the cotton fabric, the diesel droplet disappeared very fast after dropping. The fabric structure is characterized by many voids between the yarns and fibers obtained during weaving, favoring penetration and quick spreading of low viscous diesel. A chitosan layer on the fiber surface fills the gaps between them. This layer was the reason for the slight increase in the contact angle of diesel droplets on CB, and the uneven surface of CBZ decreased it again. Table 2 also includes the contact angles of the cotton fabric and CB and CBZ fabrics towards the water. Cotton fabric is hydrophilic, and water droplets disappear very fast. The contact angle of CBZ is significantly larger than that of CB, as the surface of CBZ is rougher and contains nonpolar groups.

### 3.3. BET Analysis

The surface area of the CO, CB, and CBZ samples was determined using the BET method to analyze how fabric modification can influence the adsorption properties and number of active sites. The samples were pretreated to remove any adsorbed moisture, followed by nitrogen physisorption isotherms against relative pressure at −196 °C. The calculated parameters obtained by the analysis are presented in Table 3. With the chitosan layer’s deposition on the cotton fabric’s surface, a formed film fills the pores between fibers. The reduction in porosity decreases the specific surface area of a CB composite material compared to cotton fabric (CO). The inclusion of ZnO particles in the CBZ sample again creates pores in the chitosan layer, and the specific surface area of the material increases, improving its sorption properties (Table 3). CBZ is also characterized by the highest values of the total pore volume and average pore diameter compared to materials CO and CB.

**Figure 5:**
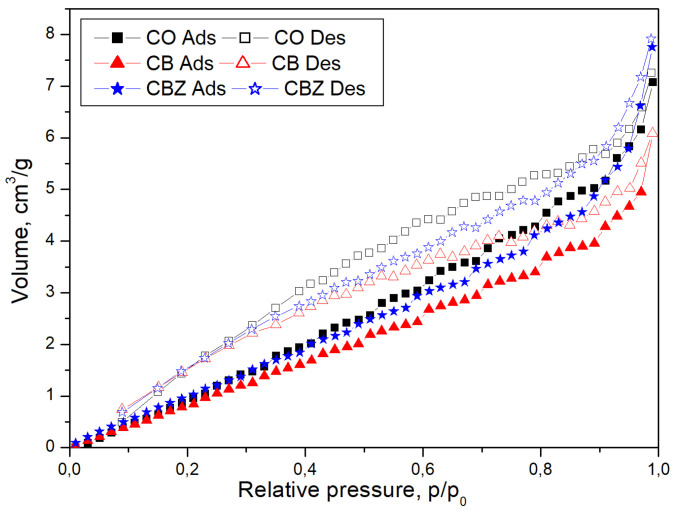
Nitrogen adsorption/desorption isotherms of the cotton fabric CO and CB and CBZ samples.

Figure 5 compares the nitrogen adsorption/desorption isotherm curves of pristine cotton fabric CO with the obtained data for CB and CBZ materials. The adsorption/desorption isotherms are approximately identical for CO and CBZ. In the case of CB, the adsorption and desorption values decrease compared to the values of the other samples, CO and CBZ, when the relative pressure values p/p0 become higher than 0.4. The isotherm data display that after the monolayer gas molecules’ deposition on the porous walls, the multilayer formation starts, followed by gas condensation at high pressure. According to the IUPAC classification, these curves can be assigned as type IV isotherms [32]. Therefore, the samples have a mesoporous structure, and their pore diameters are higher than 2 nm, as seen in Table 2. The appearance of the adsorption hysteresis with a type H3 loop in the nitrogen adsorption/desorption isotherms of the cotton fabric CO and CB and CBZ samples is characteristic of slit-shaped mesopores formed with aggregates of plate-like particles, or for the pore network consisting of macropores, which are not filled fully with liquid-like condensate [33].

The chitosan modification with the aldehydes and its interaction with the ZnO particles were analyzed by Infrared Spectroscopy (IR) and X-ray Diffraction (XRD) in our previous study [31]. The comparison of the spectra of the cotton fabric and those of the CB and CBZ showed the appearance of new bands at 1647 cm^−1^ and 1593 cm^−1^. They can be assigned to the vibrational oscillation of C=O in the amide groups and C=N in the Schiff bases due to modification and cross-linking of the chitosan with benzaldehyde and glutaraldehyde, respectively. In the IR spectrum of CBZ, the absence of the band at 1546 cm^−1^ (deformation of the N-H bond) and the decreased intensity of the band at 1377 cm^−1^ (due to the C-N of aryl azomethine) confirmed the interaction of chitosan with ZnO particles. The XRD data [31] confirmed a hexagonal crystal structure of the ZnO particles.

### 3.4. Sorption Capacity

**Figure 6:**
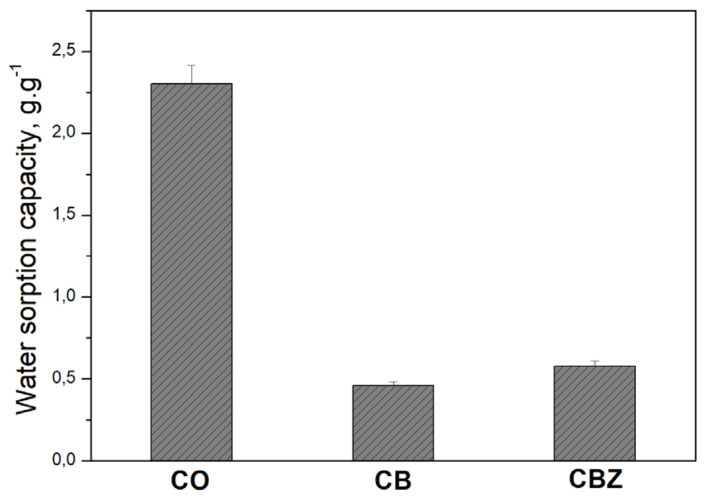
Water sorption capacity of the cotton fabric CO and samples CB and CBZ.

According to the methodology described in Section 2.4.1, the CB and CBZ composite materials’ sorption capacities were examined against water and compared with those of the pristine cotton fabric. The results presented in Figure 6 prove that the hydrophilicity significantly decreases after modification of the original cotton fabric. The very low sorption capacities of the composite textile materials (CB and CBZ) to water are evidence of their hydrophobicity. Despite the larger water contact angle of CBZ, its rough outside is responsible for the higher amount of water adsorbed compared to CB. During the experiment, the water wets the bumpy microstructures of the CBZ surface so that the formed valleys are filled with liquid.

**Figure 7:**
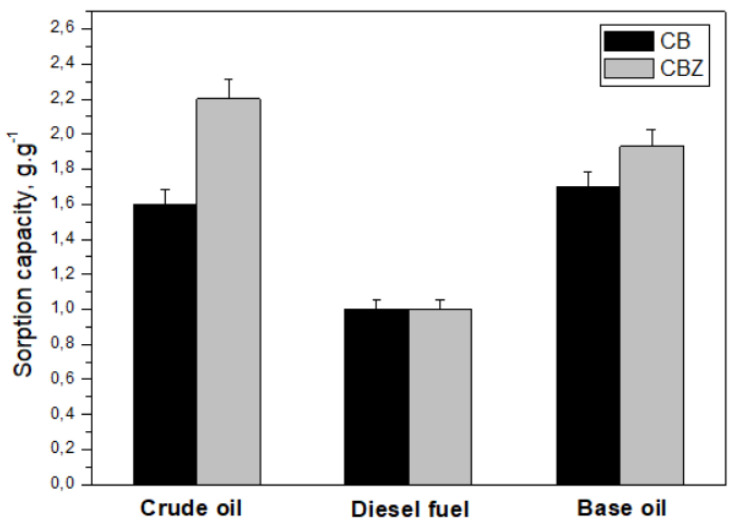
Sorption capacity of the CB and CBZ samples.

The sorption capacity of the composite materials CB and CBZ was examined against crude oil, base oil SN 150, and diesel fuel (Figure 7). The compounds typically found in crude oil as major constituents are alkanes, cycloalkanes, aromatic and polyaromatic hydrocarbons, resins, and asphaltenes. Besides alkanes and cycloalkanes, the other compounds contain one or more benzene rings. The composition of crude oil varies by many factors, while petroleum products such as base oil and diesel fuel are mixtures of fewer compounds. The quantity of aromatic compounds increases in the order of diesel fuel < base oil < crude oil [34]. The pristine cotton fabric is hydrophilic, readily adsorbs water, and sinks quickly, so its sorption capacity for investigating spills in water cannot be measured. In the CB material, the cotton fabric was coated with chitosan, which contains benzene substituents. This provides an opportunity for the π–π interaction between them and the components of crude oil and its products. The result is stronger electrostatic forces between the material and the pollutant spill, significantly improving its sorption capacity. The results in Figure 7 show that adding ZnO to the CBZ composite material’s surface improves its performance compared with the CB sample to crude oil and base oil. CBZ’s sorption capacity is best against crude oil, followed by base oil, and lowest against diesel fuel.

The base oil contains mainly unbranched alkane hydrocarbons, which are nonpolar. Compared with the other sorbates used in the study, it has the highest density and viscosity. The high viscosity and long hydrocarbon chains of the compounds in the base oil can lead to two opposite effects: the improvement of sorption capacity through better wetting of the material surface and the improved adhesion of the oil onto the fibers’ surface but hard permeation into the pores of the composite material [35]. The low values of the materials’ sorption capacity concerning diesel fuel are probably due to the low values of its density and viscosity. The low viscosity of diesel fuel, on the one hand, leads to faster diffusion to the surface of the sorbent and its retention in the pores due to capillary forces. On the other hand, it easily drips during measurements, leading to lower adsorption capacity values [36].

The sorption capacities of the developed materials in this study have been compared with the values in the literature. As displayed in Table 4, the sorption capacities of the developed materials are closely correlated with the properties of the other analogous materials reported in the scientific literature. The advantage of the CBZ material in this study is its flexibility. It can be cut with scissors, can be easily separated from water because it does not sink, can be regenerated, and, most importantly, has faster sorption abilities. The technology for its preparation is not complicated. It is prepared from cheap, non-toxic, and biodegradable raw materials. Its main components are some of the most common biopolymers, such as cellulose and chitin, converted into chitosan.

### 3.5. Regenerability of Sorbents

**Figure 8:**
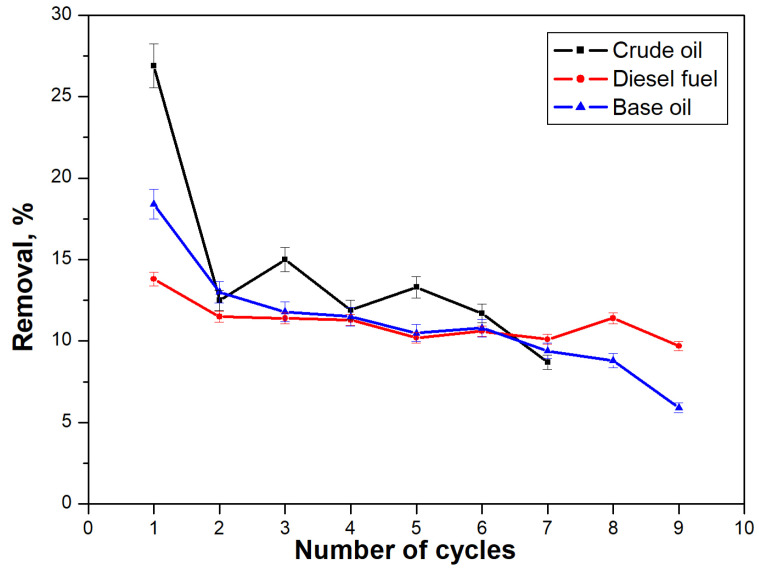
Regenerability of CBZ material.

The operational qualities of the obtained materials are determined by their ease of use, stability, and good and fast sorption capacity, and their economic efficiency depends on the possibility of their repeated use and easy regeneration, with the possibility of utilizing the separated crude oil or petroleum product. Experiments were conducted to investigate the possibility of repeated use of the CBZ material after regeneration with hexane until the entire removal of contaminants (Figure 8).

The obtained results (Figure 8) show that the complete sorption of crude oil from the water surface takes place with the least number of immersions after regeneration of the material. These results are probably due to the fact that crude oil in water remains visibly distinguishable, which facilitates the localization of the spill and the application of the sorbent to it. With diesel fuel and base oil spills, due to their transparency, it is difficult to locate the spill, especially since the amount decreases after the first few applications of the composite materials.

The highest percentage of pollutant removal is observed during the initial use of the material since the quantity and thickness of the pollutant layer are the largest, and the entire surface area of the adsorbent is in contact with the crude oil or oil products. In subsequent applications of the composite textile material, its whole surface does not come into contact with the specific contaminant. Part of it also comes into contact with the water. Nevertheless, the effectiveness of the material remains unchanged (within the range of 5%). However, CBZ proves its success in completely removing the contaminants from the water surface after regeneration.

## 4. Conclusions

Coating cotton fabric with chitosan, containing benzene rings as substituents and cross-linked with glutaraldehyde, increases its hydrophobicity. Compared with pristine cotton material, the obtained CB fabric does not sink in water. The possibility of floating makes the fabric suitable for wiping oil spills. The integration of ZnO particles in the film mentioned above increases the fabric’s sorption capacity due to the specific pore rough surface formation. It enhances the surface hydrophobicity, as ZnO particles form complexes with the hydroxyl and amino groups of the chitosan layer. SEM, AFM, and BET analyses established the change in surface characteristics after the fabrics were modified. The contact angle measurement to water, crude oil, and its products confirmed the improved hydrophobic and oleophilic properties of the CBZ fabric compared with CB. This specific rough surface is the reason for the better sorption properties of the CBZ material towards crude oil and its products. The contact angle determination also confirmed the ability of new materials to adsorb contaminants. The highest sorption capacity, in the CBZ sample, is shown towards crude oil, and the lowest is for diesel fuel. These results are probably due to diesel fuel’s lower density and viscosity values. The possible regeneration and multiple reuse of CBZ define its application as effective and economically sustainable. Future research will aim to increase the materials’ hydrophobic and oleophilic properties, improving their performance as environmentally friendly and sustainable materials applied successfully as sorbents for cleaning up oil spills.

## Figures and Tables

**Table 1:** Characteristics of the used crude oil and petroleum products.

Sorbate	Density, g/cm^3^ (15 °C) EN ISO 3675 [29]	Kinematic Viscosity, mm^2^/s (40 °C) EN ISO 3104 [30]
Crude oil	0.830	12.3
Diesel fuel	0.827	2.9
Base oil SN 150	0.864	34.3

**Table 2:** Contact angles of pollutants on the sorbents.

Sample	Contact Angles, [°]			
	Crude Oil	Diesel Fuel	Base Oil	Water [31]
CO	71.13 ± 1.05	26.00 ± 2.54	83.49 ± 1.05	0
CB	63.60 ± 1.82	28.93 ± 0.23	69.84 ± 0.25	77.13 ± 0.91
CBZ	60.78 ± 2.21	25.69 ± 0.22	68.29 ± 1.61	101.76 ± 1.14

**Table 3:** Characteristic of the cotton fabric and the textile composite materials.

Sample	Total Pore Volume cm^3^/g	Average Pore Diameter nm	BET Specific Surface Area m^2^/g
CO	0.01097	4.061	10.80
CB	0.00944	5.496	6.870
CBZ	0.01202	6.619	7.265

**Table 4:** Comparison of oil sorption capacities from this study and the reported literature.

Sorbent	Oil Type	Sorption Capacity	Time	Ref.
Lauric acid-treated oil palm leaves	Crude oil	1.20 g/g	20 min	[37]
Hydrogel of chitosan based on polyacrylamide (HCP)	Crude oil	2.30 g/g	200 min	[38]
Hydrophobic aerogel (HA)	Crude oil	2.8 g/g	180 min	[39]
Orange peel Thermally modified orange peel	crude oil, diesel oil, kerosene, and gasoline	3–5 g/g 3.5–7 g/g	30 min	[40]
Woven cotton, modified with poly(N,N-dimethylaminoethyl methacrylate) and poly(dimethylsiloxane)	hexadecane	2.2 g/g	90 min	[41]
Acetylated Siamese senna seed pods	Crude oil	6.83 g/g	15 min	[42]
Polyurethane foam cellulose composite	Engine oil	3.91–12.49 g/g	5 h	[43]
Natural rubber	Crude oil	6–7 g/g	15 min	[44]
DPF DPF-CA/ZnO	Diesel oil	26.5 mg/g 66.4 mg/g	60 min	[45]
CBZ	Crude oil, Diesel oil Base oil	2.2 g/g 1.0 g/g 1.93 g/g	10 min	Current study

## Data Availability

The original contributions presented in the study are included in the article/Supplementary Material, further inquiries can be directed to the corresponding authors.

## Supplementary Materials

The following supporting information can be downloaded at https://www.mdpi.com/article/10.3390/ma18051146/s1, Figure S1. The contact angles of the petroleum, diesel fuel, and base oil SN 150 on the pristine cotton fabric and composites CB and CBZ.
