# Supplementary material for: Application of Textile Composite Materials as a Sorbent for Cleaning Up Oil Spills
Source: Materials (Basel). 2025 Mar 4;18(5):1146. doi: 10.3390/ma18051146 (PMC11901613; doi:10.3390/ma18051146)
Supplement: Supplementary file 1 [file materials-18-01146-s001.zip › materials-3481315-supplementary.pdf]

Supplementary Information

Application of Textile Composite Materials as a Sorbent for Cleaning Up Oil Spills

Daniela Angelova *, Desislava Staneva *, Daniela Atanasova and Vesislava Toteva

**CO (cotton)**		
Petroleum	Base oil	Diesel
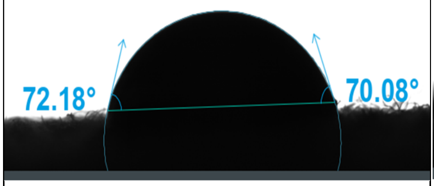	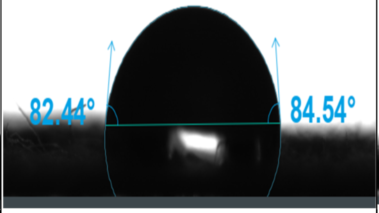	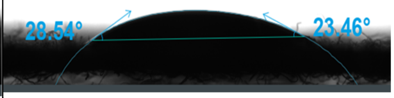
Mean CA = 71.13°	Mean CA = 83.49 °	Mean CA = 26°
**CB**		
Petroleum	Base oil	Diesel
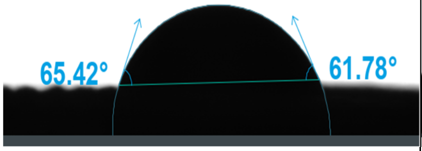	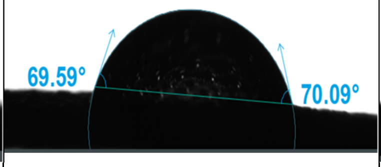	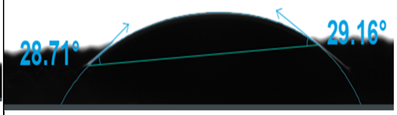
Mean CA = 63.60 °	Mean CA = 69.84 °	Mean CA = 28.93 °
**CBZ**		
Petroleum	Base oil	Diesel
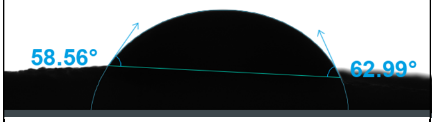	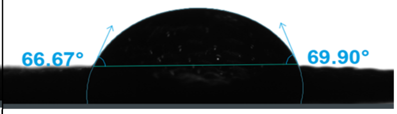	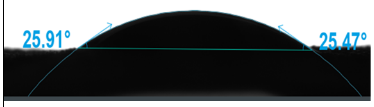
Mean CA = 60.78 °	Mean CA = 68.29 °	Mean CA = 25.69 °

Figure S1. The contact angles of the petroleum, diesel fuel, and base oil SN 150 on the pristine cotton fabric and composites CB and CBZ
